# Dentin Sialophosphoprotein (DSPP) Gene-Silencing Inhibits Key Tumorigenic Activities in Human Oral Cancer Cell Line, OSC2

**DOI:** 10.1371/journal.pone.0013974

**Published:** 2010-11-12

**Authors:** Rajeshree Joshi, Amany Tawfik, Nneka Edeh, Veronica McCloud, Stephen Looney, Jill Lewis, Stephen Hsu, Kalu U. E. Ogbureke

**Affiliations:** 1 Department of Oral Biology, School of Dentistry, Medical College of Georgia, Augusta, Georgia, United States of America; 2 Department of Pathology, School of Medicine, Medical College of Georgia, Augusta, Georgia, United States of America; 3 Department of Oral Health and Diagnostic Sciences, School of Dentistry, Medical College of Georgia, Augusta, Georgia, United States of America; 4 School of Graduate Studies, Medical College of Georgia, Augusta, Georgia, United States of America; 5 Department of Biostatistics, School of Graduate Studies, Medical College of Georgia, Augusta, Georgia, United States of America; Karolinska Institutet, Sweden

## Abstract

**Background:**

We determined recently that dentin sialophosphoprotein (DSPP), a member of the SIBLING (Small integrin-binding ligand N-linked glycoproteins) family of phosphoglycoproteins, is highly upregulated in human oral squamous cell carcinomas (OSCCs) where upregulation is associated with tumor aggressiveness. To investigate the effects of *DSPP*-silencing on the tumorigenic profiles of the oral cancer cell line, OSC2, short-hairpin RNA (shRNA) interference was employed to silence *DSPP* in OSC2 cells.

**Methodology/Principal Findings:**

Multiple regions of DSPP transcript were targeted for shRNA interference using hDSP-shRNA lentiviral particles designed to silence DSPP gene expression. Control shRNA plasmid encoding a scrambled sequence incapable of degrading any known cellular mRNA was used for negative control. Following puromycin selection of stable lines of *DSSP*-silenced OSC2 cells, phenotypic hallmarks of oral carcinogenesis were assayed by western blot and RT-PCR analyses, MTT (cell-viability), colony-formation, modified Boyden-Chamber (migration and invasion), and flow cytometry (cell-cycle and apoptosis) analyses. *DSPP*-silenced OSC2 cells showed altered cell morphology, reduced viability, decreased colony-formation ability, decreased migration and invasion, G0/G1 cell-cycle arrest, and increased tumor cell sensitivity to cisplatin-induced apoptosis. Furthermore, MMP-2, MMP-3, MMP-9, VEGF, Ki-67, p53, and EGFR were down-regulated. There was a direct correlation between the degree of *DSPP*-silencing and MMP suppression, as indicated by least squares regression: MMP-2 {(y = 0.850x, p<0.001) (y = 1.156x, p<0.001)}, MMP-3 {(y = 0.994x, p<0.001) (y = 1.324x, p = 0.004)}, and MMP-9 {(y = 1.248x, p = 0.005, y = 0.809, p = 0.013)}.

**Conclusions/Significance:**

*DSPP*-silencing in OSC2 cell decreased salient hallmarks of oral tumorigenesis and provides the first functional evidence of a potential key role for DSPP in oral cancer biology. The down-regulation of MMP-2, MMP-3, MMP-9, p53 and VEGF in *DSPP*-silenced OSC2 cells provides a significant functional/molecular framework for deciphering the mechanisms of DSPP activities in oral cancer biology.

## Introduction

Dentin sialophosphoprotein (DSPP) is a member of the SIBLING (Small Integrin-Binding LIgand N-linked Glycoprotein) family of extracellular matrix glycophosphoproteins [1]. Other members of the family are bone sialoprotein (BSP), dentin matrix protein 1 (DMP1), dentin sialophosphoprotein (DSPP), osteopontin (OPN), and matrix extracellular phosphoglycoprotein (MEPE) [1]. Expression of the SIBLINGs was originally thought to be limited to bone and teeth where they function to facilitate dentin and bone matrix mineralization [1]–[3]. Recent reports however indicate that the SIBLINGs are also present in non-mineralizing metabolically active ductal epithelial cells of the salivary glands, nephrons, and eccrine sweat glands where their function may be associated with the repair of pericellular and extracellular matrix (ECM) proteins damaged by free radical generated through intense metabolic activity [4]–[6].

Earlier reports have identified the up-regulation of some members of the SIBLINGs in various cancers, including breast, lung, and prostate cancers [7]. OPN however is the SIBLING for which there is unequivocal evidence for its role in many of the steps of cancer development and progression [6]–[8]. Accumulating data are beginning to implicate other family members, notably BSP and DSPP, with roles in specific stages of tumor progression including cell growth, adhesion, migration, and/or metastasis [6]–[8]. We recently reported the up-regulation of BSP, DSPP, and OPN, in human oral squamous cell carcinomas (OSCCs) [9] and in some human oral epithelial dysplasia (OEDs) [10]. These reports indicate that DSPP is highly up-regulated in poorly differentiated and histologically aggressive OSCC [9]. Significantly, OEDs expressing DSPP with or without BSP exhibited a 4-fold propensity for transition to OSCC compared to OEDs expressing BSP alone, suggesting that DSPP expression increased the risk of local primary OSCC while BSP expression decreased such risk [10]. However, there currently are no data on the functional and mechanistic role of DSPP in oral cancer development and progression.

In the present study designed to establish functional correlations between DSPP expression and the biologic behavior of OSCC, short hairpin RNA (shRNA) interference was used to produce silencing of *DSPP* in OSCC cell line OSC2. *DSPP*-silenced OSC2 cells were then evaluated to determine the extent to which silencing suppresses or abrogates key malignant phenotypic characteristics of OSC2 cells using various standard *in vitro* techniques.

## Results

### DSPP is upregulated in oral cancer cell lines, OSC2 and SCC25, and in dysplastic oral epithelial cell line, DOK

**Figure 1:**
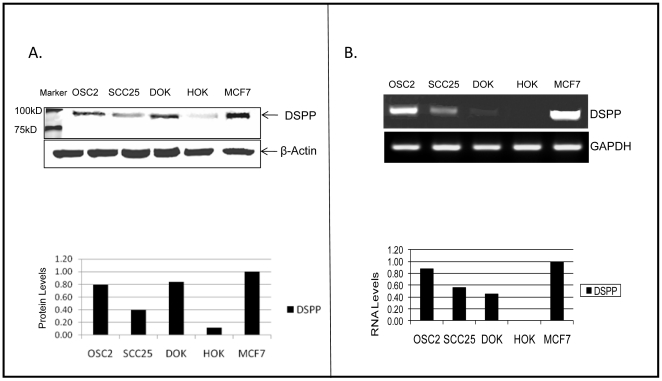
DSPP upregulation in oral-cancer cells, OSC2 and SCC25, and the dysplastic oral keratinocyte cells, DOK. (A) Western blot (WB) and densitometric analyses show significant upregulation of DSPP in OSC2, SCC25, and DOK cells compared with HOK negative controls. There is a basal (<10%) level of DSPP expression in primary HOK cells. MCF7 cell line known to express DSPP was used as positive control. Normalization was with β-actin. (B) Semiquantitative RT-PCR analysis shows DSPP-mRNA expression in OSC2, SCC25, and DOK cells consistent with WB results in (A) with undetectable DSPP-mRNA levels in HOK cell. Normalization was with GAPDH. Cells used in study: **OSC2**, a human OSCC cell line derived from regional cervical lymph node metastasis of a primary human tongue squamous cell carcinoma; **SCC25**, a primary tongue squamous cell carcinoma; **DOK**, a human oral epithelial dysplastic cell line derived from dorsal tongue; and **MCF7** (acronym of Michigan Cancer Foundation – 7), a human breast cancer cell line isolated from a 69-year-old Caucasian female.

OSC2 and SCC25 are human OSSC cell lines derived from regional cervical lymph node metastasis of a primary human tongue squamous cell carcinoma, and a primary tongue squamous cell carcinoma, respectively [11]–[13]. DOK is a human oral epithelial dysplastic cell line derived from dorsal tongue [14]. To evaluate the endogenous expression levels of DSPP protein and mRNA in OSC2, SCC25, and DOK cells, western blot and semiquantitative-reverse transcriptase (RT)-PCR analyses were carried out on whole-cell lysates and total RNA extracts, respectively, as described in the Material and Methods. Positive and negative controls consisted of whole-cell lysates and total RNA extracts from MCF-7 cell (derived from mammary adenocarcinoma) and HOK cells (derived from primary culture of human oral keratinocytes), respectively. DSPP is significantly upregulated in OSC2, SCC25, and in DOK compared with HOK cells at the translational (Figure 1A) and transcriptional (Figure 1B) levels. These *in vitro* findings are consistent with the results of our recent studies indicating the up-regulation of DSPP in OSCC from patients, and its complete absence in normal oral mucosal epithelium [9].

### Transient DSP-shRNA transfection results in altered morphology of OSC2 cells

**Figure 2:**
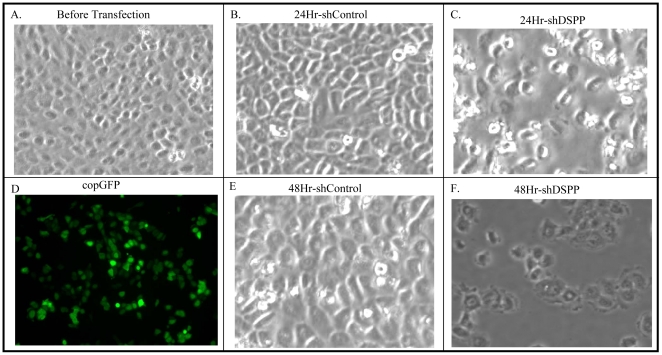
Transient lentiviral-mediated DSP-shRNA knockdown results in altered morphology of OSC2 cells. (A) OSC2 cells prior to lentiviral-transduced transfection with DSP-ShRNA exhibiting characteristic morphology of viable epithelial tumor cells in culture. (B, E) Scrambled (control) sequence-transfected OSC2 cells at 24- and 48 h exhibiting viable epithelial cells and morphology comparable to (A). (C, F; illustrative areas) DSP-shRNA transfection OSC2 cells exhibiting significantly increased number of cells with loss of cell-cell contact, more rounded and irregular outline, prominent nuclear blebbing, and cellular disintegration consistent with those of effete and dying cells at 24- and 48-h, respectively. (D) copGFP transfected OSC2 cells confirm very high transfection efficiency (green fluorescence).

Having established baseline expression levels of DSPP in two OSCC cell lines, we proceeded to investigate how stable *DSPP*-silencing via lentiviral-mediated small hairpin RNA (shRNA) interference affects the tumorigenic profiles of OSC2 cells. We chose OSC2 cells over their SCC25 counterpart because of the demonstrably higher expression levels of DSPP in OSC2 cells at both the protein and mRNA levels (Figure 1) and also because OSC2 cells exhibit very strong invasive phenotype as evidenced in experimental nude mouse models [11]–[13]. In order to assess the immediate effects of DSP-shRNA transfection on OSC2 cell morphology, transfected cells along with controls were photographed at 24- and 48-hr post-transfection (transient stage) prior to commencement of puromycin selection of stably transfected cells. As shown in Figures 2C and 2F (illustrative areas photographed), DSP-shRNA transfected OSC2 cells exhibited a by far greater number of cells with considerable loss of the cell-cell contact and a more ovoid and irregular outline compared with non-transfected OSC2 cells (Figure 2A) or scrambled sequence controls (Figures 2B, 2E). Fluorescence microscopy of transiently transfected coGFP-shRNA clones showed a strong green fluorescence indicative of high percentage transfection efficiency (Figure 2D). We proceeded to establish stable DSP-shRNA-silenced OSC2 cells via puromycin selection in order to investigate the extent of any changes in other salient phenotypic hallmarks of OSCC.

### Generation of a stable *DSPP*-silenced OSC2 cell line

**Figure 3:**
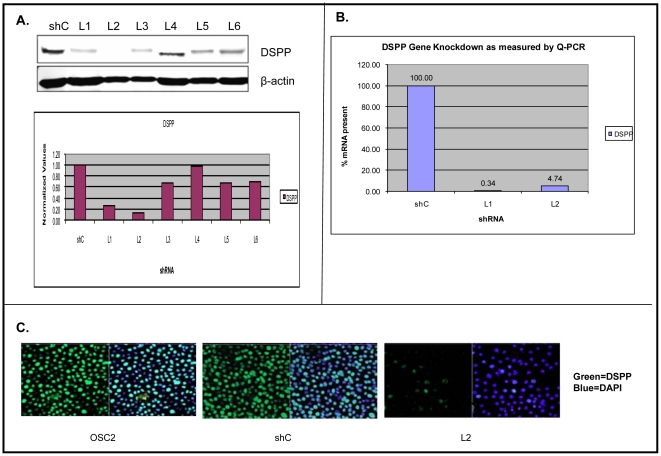
Generation of stable *DSPP*-silenced OSC2 cell lines. Stable lines selection of lentiviral-transduced shRNA was accomplished via puromycine selection. (A) WB and corresponding densitometric analyses of level of DSPP-silencing following selection of six stable lines show level of silencing ranging from ∼5% (line [L] 4) to ∼95% (L2) compared to scrambled sequence (shC) control. (B) qRT-PCR analysis show significantly reduced DSPP-mRNA in DSPP-silenced L1 (>90%) and L2 (>95%) cells (exhibiting the most knockdown on WB) compared to shC control. (C) Immunfluorescence confocal microscopy verifies DSPP-silencing as significantly reduced DSPP signal (green fluorescence) in L2.

Stable lines of DSP-silenced and scrambled sequence controls were selected by means of puromycin antibiotics as described in Materials and Methods. The effectiveness of DSP-shRNA lentiviral construct in stably silencing DSPP in OSC2 cells was verified by western blot and quantitative real-time PCR (qRT-PCR) analyses. As shown in Figure 3, western blot and densitometric analyses of six different DSP-shRNA transfected OSC2 stable lines showed DSPP silencing ranging from ∼5% [Line (L) 4] to ∼95% L2 compared with stable shRNA-scrambled sequence (shC) control (Figure 3A). These results were further confirmed by immunofluorescence confocal microscopy showing significantly reduced DSPP signal (green fluorescence) in L2 stable lines compared with control (Figure 3C).

In parallel, we verified the effectiveness of DSP-shRNA in depleting DSPP mRNA in lentivirally infected cells by qRT-PCR using L1 and L2 exhibiting the most knockdown of the six lines (see Figure 3A) on western blot. The results indicate significantly reduced DSPP mRNA levels in L1 (>99%) and L2 (>95%) compared with shRNA shC control line (Figure 3B). The C_T_ values obtained from qRT-PCR analysis of the relative DSPP mRNA levels in shC control and *DSPP*-silenced L1 and L2 were normalized with the housekeeping gene GAPDH. This shRNA-mediated effect was specific, as GAPDH levels did not differ significantly amongst the DSP-shRNA transfected cells and controls.

### *DSPP*-silencing reduces p53, Ki-67 and PCNA, and inhibits cell proliferation and colony-formation in OSC2 cells

**Figure 4:**
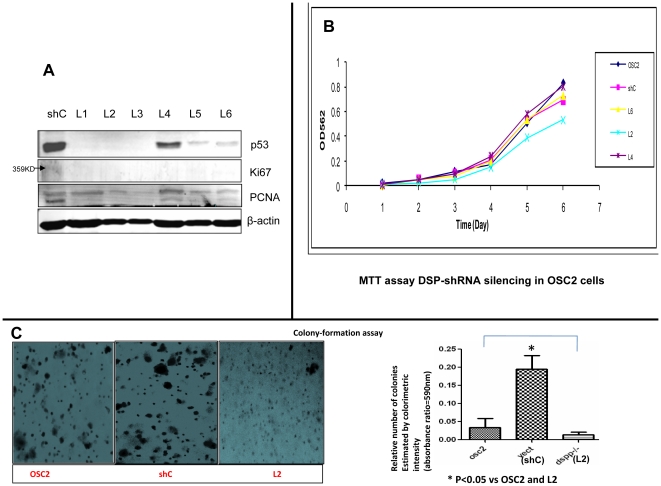
*DSPP*-silencing results in dowregulation of Ki-67, PCNA, p53, and inhibition of cell proliferation and colony-formation. (A) WB of ki-67, PCNA, and p53 following *DSPP*-silencing in OSC2 cells show significant downregulation for each of these proteins. (B) The proliferation status of *DSPP*-silenced OSC2 cells compared to parental OSC2 and shC control carried out via MTT assay shows decreased cell proliferation of 53% for L2 (demonstrating the most degree of silencing) compared with <5% for L4 (least silencing), and ∼30% for L6 (moderate silencing) and shC control (p<0.05; n = 3). (C) L2 cells formed smaller and significantly fewer (<25%) colonies in soft agar compared with parental OSC2 and shC control cells (*p<0.05).

The effects of *DSPP*-silencing on the levels of proliferative marker, Ki-67, PCNA, and the cell cycle regulator p53 were analyzed by western blot for all six *DSPP*-silenced OSC2 stable lines. Compared with ShC control line, the levels of Ki-67, PCNA, and p53 were significantly reduced, indicating that DSPP may enhance proliferation in oral cancer cells via mechanisms involving p53, Ki-67, and PCNA (Figure 4A).

In order to determine the extent to which *DSPP* silencing affects the proliferation of OSC2 cells, we carried out MTT assays for three of the six *DSPP*-silenced stable lines that included L2 demonstrating the highest degree of *DSPP*-silencing, L4 demonstrating the least silencing, and L6 demonstrating moderate silencing. Compared with shC control and parental OSC2 cell lines, silencing of *DSPP* was associated with an average of 53% decrease in cell proliferation at day 6 in L2 (Figure 4B; P<0.05; n = 3). With respect to the ability to form colonies, L2 cells formed colonies in soft agar that were considerably smaller and fewer than those from shC controls, and from parental OSC2 cells (Figure 4C). The number of colonies formed in L2 cells was significantly reduced (<25% of parent OSC2 cells and about 20% of shC control) as shown in Figure 4C bar graph. These results suggest a significant role for DSPP in oral cancer cell growth and proliferation. L2 cells demonstrating the most significant degree of *DSPP*-silencing of all six lines were employed for investigations of the effects of silencing on migration and invasion, and on cell-cycle activities.

### *DSPP*-silencing decreases OSC2 migration and invasion

**Figure 5:**
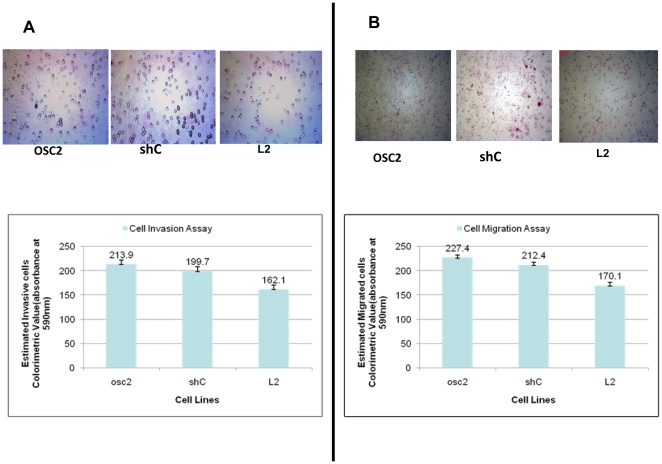
*DSPP* silencing decreased OSC2 migration and invasion. (A, B) Modified Boyden-Chamber experiment shows that *DSPP*-silencing in (L2) decreased invasion (A) and migration (B) of OSC2 cells by ∼25%, respectively, compared to shC control and parental OSC2 cells (for each comparison using Dunn methods of multiple comparisons).

As described in the Material and Methods section, we carried out invasion and migration assays using modified Boyden Chamber experiments in order to determine the extent to which *DSPP* silencing affects migration and invasion of OSC2 cells. As shown in Figure 5, silencing of *DSPP* (L2) decreased invasion (Figure 5A) and migration (Figure 5B) of OSC2 cell by 25% in each case, compared with shC control and parental OSC2 cells (p<0.05 for each comparison using Dunn method of multiple comparisons). Based on these findings we hypothesize that DSPP plays a significant role in migration and invasiveness within OSCC microenvironment to at least aid local spread of tumor. Furthermore, the results of these in vitro assays allow us to speculate that DSPP may play a role in distant site metastasis of primary OSCCs.

### Stable *DSPP*-silencing in OSC2 cells induces G_0_/G_1_ arrest

**Figure 6:**
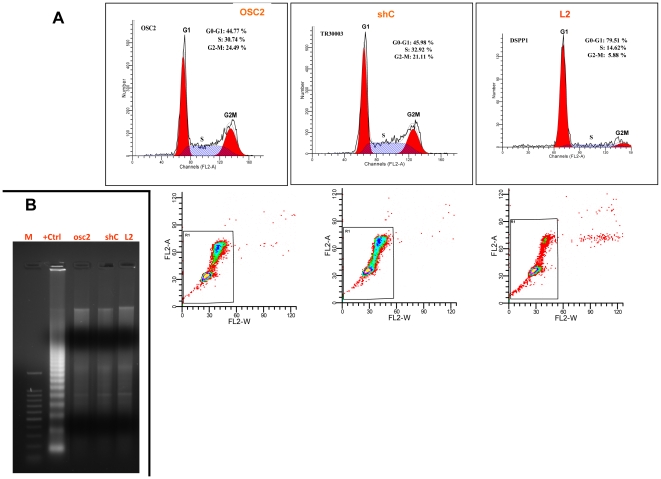
*DSPP*-silencing reduces G_0_/G_1_ arrest in OSC2 cells. (A) Flow cytometric analysis shows proportion of DSPP-silenced L2 cells in G_0_/G_1_ phase significantly increased (79.51%) compared with parental OSC2 (44.7%) and shC controls (45.98%). (B) Conversely, DNA laddering experiments shows no significant difference in rate of apoptosis between L2 cells and controls. This result indicates that *DSPP*-silencing alone does not increase the rate of apoptosis in OSC2 cells.

To verify the effects of *DSPP* suppression on OSC2 cell cycle activities, flow cytometric analysis of *DSPP*-silenced OSC2 stable line, L2, was carried out. The proportion of L2 cells in the G_0_/G_1_ phase was significantly increased compared with that of shC control and that of parental OSC2 cells (Figure 6). As shown in Figure 6A, 79.51% of L2 cells accumulated in the G_0_/G_1_ phase in contrast with 44.77% and 45.98 of parental OSC2 and shC controls, respectively. The proportions of cells in the S and G_2_/M phases of L2 cells were 14.62% and 5.88%, respectively, compared with 30.74% (S) and 24.49% (G_2_/M) for parental OSC2 cells, and 32.92% (S) and 21.11% (G_2_/M) for shC control cells. However, there were no detectable differences in apoptotic rate between L2 cells and shC controls as further confirmed by DNA laddering experiments (Figure 6B), indicating that *DSPP*-silencing alone does not increase the apoptosis rate in OSC2 cells.

### Stable *DSPP*-silencing sensitized OSC2 cells to cisplatin-induced apoptosis

Head and neck squamous cells carcinomas (HNSCC), including OSCCs, develop acquired or intrinsic resistance to cisplatin-based combination chemotherapy [15], [16]. It has been suggested that the mechanism of interference with cisplatin-induced apoptosis in HNSCCs is by upregulation of EGFR [15]. We therefore sought to determine the effect of *DSPP*-silencing on EGFR expression as well as the extent of the effects of *DSPP*-silencing on response of OSC2 cells to cisplatin-induced apoptosis.

**Figure 7:**
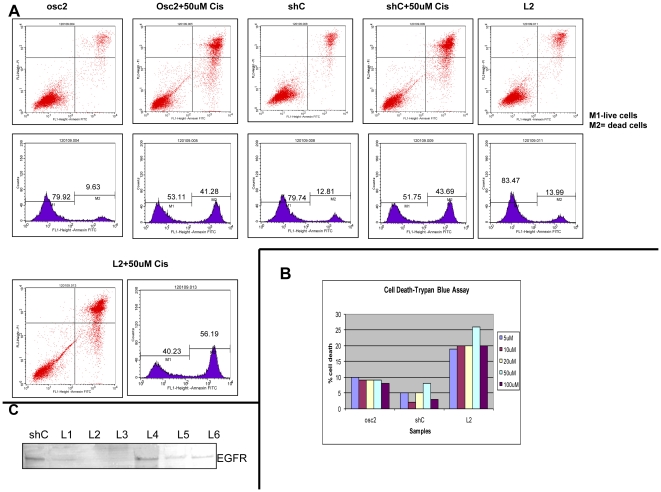
*DSPP*-silencing sensitizes OSC2 cells to cisplatin-induced apoptosis. (A) Treatment of *DSPP*-silenced L2, parental OSC2, and shC control cells with 50 uM for 24 h followed by Annexin V/FITC flow cytometry analysis show a 56.19% increase in apoptotic rate (measured by sub-G1 cellular DNA content) for L2 cells compared with 41.28% in parental with cisplatin and OSC2 and 43.69% in shC control with cisplatin. (B) Trypan blue staining bar chart quantitation indicates comparable apoptotic rates to that in (A). (C) WB showing significant down-regulation of EGFR in *DSPP*-silenced OSC2 cells, including L2. Symbols: Cis =  cisplatin; L2 =  *DSPP*-silenced OSC2 cells line 2; OSC2 =  parental (without knockdown) osc2 cells, M1 = live cells; M2 =  deal cells.

Following treatment of *DSPP*-silenced OSC2 (L2) cells and controls (parental OSC2 and shC) with various doses of cisplatin in a time-course experiment as described in the Material and Methods section, rate of apoptosis in L2 cells was analyzed by Annexin V/FITC flow cytometry, and compared with rates in controls. With almost 100% of cells gated and FACS sorted, Figure 7A shows that the apoptotic cell fraction was significantly increased from 41.28% in parental OSC2 cells (and 43.69% in shC controls) treated with 50 uM cisplatin for 24 hrs to 56.19% in L2 cells treated with equal dose of cisplatin. On the other hand, the apoptotic cell fractions without cisplatin for OSC2 (9.63%), ShC control (12.81%), and L2 cells (13.99%) were not significantly different. Similarly, results of trypan blue staining indicate equally significant increase in the rate of apoptosis in L2 cells treated with cisplatin compared with shC control and parental OSC2 cells treated with cisplatin at various doses (Figure 7B).

Furthermore, the level of EGFR was significantly reduced in L2 cells compared to shC controls, suggesting that EGFR inhibition is required for cisplatin-induced apoptosis in OSC2 cells (Figure 7C). Collectively, these results suggest that DSPP silencing in OSC2 cells, while resulting in G_0_/G_1_ arrest (see Figure 6A) and therefore reduced proliferative activity, does not increase apoptosis; however, DSPP suppression may significantly enhance the sensitivity of OSC2 cells to cisplatin-induced apoptosis via mechanism involving the downregulation of EGFR.

### Stable *DSPP*-silencing in OSC2 cells suppresses MMP-2, MMP-3, and MMP-9 expressions

Earlier reports have identified specific interactions between some members of the SIBLING family of proteins with specific matrix metalloproteinases (MMPs). Specifically, BSP partners with proMMP-2, OPN with proMMP-3, and DMP-1 with proMMP9 in order to activate the respective proteases [4], [17]. However, the MMP partner(s) to DSPP and MEPE, if any, have not been identified. Nevertheless, we sought to determine the expression status of these three known SIBLING-partnering MMPs in all six generated *DSPP*-silenced OSC2 lines.

**Figure 8:**
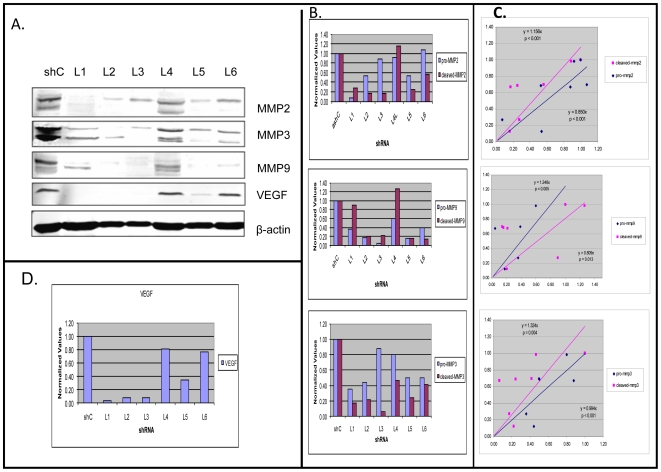
*DSPP*-silencing downregulates SIBLING-partnering MMPs in OSC2 cells. (A) WB and (B) corresponding densitometric analyses show significant down-regulation of pro- and activated MMP-2, MMP-3, and MMP-9 in all but one (L4) of the six *DSPP*-silenced stable lines. (C) Least square regression analyses show significantly reduced levels of pro-and activated MMP-2 {(y = 0.850x, p<0.001) (y = 1.156x, p<0.001)}; MMP-3 {(y = 0.0.994x, p<0.001) (y = 1.324x, p = 0.004)}, and MMP-9 {(y = 1.248x, p = 0.005, y = 0.809, p = 0.013)}, and a direct correlation between the degree of *DSPP*-silencing and MMP suppression; however, the level of MMP suppression did not differ significantly between pro- and cleaved forms. (D) Densitometric analysis of WB of VEGF levels showed down-regulation ranging from <5% (L4) to 86% (L2) with the level of down-regulation directly proportional to the degree of *DSPP*-silencing.

As shown by western blot and densitometric analyses, levels of both pro- and activated MMP-2, MMP-3, and MMP-9 levels were reduced in all but one of the six *DSPP*-silenced OSC2 lines compared with shC controls (Figures 8A, 8B). Equally significant is a direct correlation between the degree of *DSPP*-silencing and MMP suppression, as indicated by least squares regression analysis through the origin: MMP-2 {(y = 0.850x, p<0.001) (y = 1.156x, p<0.001)}, MMP-3 {(y = 0.994x, p<0.001) (y = 1.324x, p = 0.004)}, and MMP-9 {(y = 1.248x, p = 0.005, y 0.809, p = 0.013); Figure 8C)}. However, the level of MMP suppression did not differ significantly between pro- and cleaved forms. Similarly, decrease in VEGF levels in stable *DSPP*-silenced lines ranged from <5% (L4) to 86% (L2) with the level of suppression directly proportional to the degree of *DSPP*-silencing (Figure 8D), suggesting a dose-dependent relationship between DSPP expression and angiogenic activity in OSCCs. Collectively, these results suggest a regulatory role for DSPP in the upregulation and activation of the SIBLING-partnering MMPs as well as angiogenesis in oral cancer.

### *In vivo* anti-tumor effects of *DSPP*-silencing on OSC2 cells

The anti-tumor effect of DSPP-silencing in OSC2 cells was tested in two Balb/c nude mice implanted subcutaneously with DSPP-silenced OSC2 cells (L2 lines) on the left flank, while the scrambled sequence control (shC lines) was implanted subcutaneously on the right flank. As shown in the Supplementary Figure S1, a trend towards slowed tumor development and growth resulting in an overall smaller tumor volume was observed for L2 tumor compared with shC control tumors over a period of 6 weeks [Figures S1A and S1G (scatter plot]. Histologic evaluation of hematoxylene and eosin (H&E) sections showed well differentiated and aggressive squamous cell carcinoma associated with the shC (control) derived tumors (Figure S1B) compared with the less-differentiated L2 derived tumors (Figure S1C). In addition, L2 derived tumors tended to form in small shrinking islands with more visible tumor necrosis than shC derived tumors (Figure S1C). Significantly, immuostain for DSPP verified DSPP reduction in L2 derived tumors (Figure S1E) compared with high DSPP level in shC derived tumors (Figure S1D). Figure S1F is a representative pre-immune IgG negative control. These results suggested that the reduced tumorigenic hallmarks of OSCC following *DSPP*-silencing observed in the *in vitro* experiments described above are reproducible *in vivo*.

## Discussion

This study, aimed at gaining insights into the functional role of DSPP in the biology of oral cancer, was designed as a logical sequel to our earlier reports indicating that DSPP is highly upregulated in aggressive human OSCCs [6] and in those OEDs with significantly high propensity for transition to invasive OSCC [10]. To the best of our knowledge, our present report represents the first demonstrating the upregulation of DSPP in two oral cancer cell lines compared to, at best, basal level in primary normal oral keratinocytes (NOK). More significantly, this is the first report on a study that investigated the effects of *DSPP*-silencing on the tumorigenic profiles of an oral cancer cell line.

*DSPP*, the largest member of the SIBLING gene family, encodes a ∼1300-amino acid protein post-translationally cleaved into two major proteins, dentin sialoprotein (DSP) and dentin phosphoprotein (DPP, also called phosphorin) [2], [18]–[20]. The expression of DSP and DPP was originally thought to be limited to dentin (and odontoblasts), where they play a major role in mineralization, with DPP being the more abundant of the two proteins [2], [19], [21]. However, subsequent studies showed that lower level of DSPP expression is present in bone [1]–[3]. Recent studies also have shown significant levels of expression of DSPP (and other members of the SIBLING family) in metabolically active ductal epithelial cells [4]–[6], and its upregulation in a subset of breast, oral, lung, and prostate cancers [6]–[9]. Because DSPP contains the highly conserved MQXDPP motif that has been shown in DMP1 to be involved in proteolytic processing, it has been hypothesized that the same tolloid-related proteases (particularly BMP1) cleave DSPP into DSP and DPP [19], [20]. Proteolytic processing of DSPP by MMP-2, MMP-20, and kallikrein-related peptidase 4 has been reported [19].

The monoclonal antibody, LF-Mb21, used in this study recognizes both the full-length (DSPP) and the DPP portion but not the DSP. The 97 kDa protein band in Figure 1A is indicative of a cleavage product, DPP, but lower molecular weight bands that would represent DSP were not present. Furthermore, molecular weight band approximating that of the full length DSPP was absent. Following silencing with a construct design that targeted the full length *DSPP*, only very low levels of the 97KD band was seen in most of the stable lines, indicating the silencing of *DPP* and full length *DSPP*. While it is conceivable that our knockdown strategy also might have reduced DSP levels, we cannot determine, based on our present data, whether or not the various effects of knockdown on the tumorigenic profile of OSC2 cells described were the result of decreased levels of DPP, DSP, or both (or even intact DSPP). It is nonetheless highly suggestive at this point that, in the least, DPP or its modified forms may play a role in the tumorigenic regulatory activities of oral cancer cells. Further studies, including strategies (e. g. adding some furin inhibitors; see Ref. [20]) that causes the cells to secrete only full length DSPP that could be used as a standard will be required for subsequent inquiries addressing any specific mechanistic role for DPP, DSP or the full length DSPP in the biology of oral cancers.

Reviews of the specific genes products profiled in this study, following *DSPP* silencing, indicate that none of them have, until now, been associated with the potential role of DSPP in oral cancer. As a secreted protein DSPP represents an ideal target for a shRNA-mediated silencing, and by analyzing the functional effects of *DSPP*-silencing in OSC2, we show that *DSPP*-silencing down-regulates key proteins involved in tumor cell proliferation, angiogenesis, and local invasion. Specifically, our data show that silencing of *DSPP* in oral cancer cells is associated with significant down-regulation of the SIBLING-partnering MMPs, VEGF, p53, and the cell proliferation markers Ki-67 and PCNA. Up-regulations of MMP-2, MMP-3, MMP-9, VEGF, and p53 with levels correlating with outcome and prognostic parameters such as advanced disease stage, invasion, and metastasis have previously been associated with oral cancer [22]–[24]. Thus, our results indicating a significant down-regulation of MMP-2, MMP-3, MMP-9, VEGF, and p53 following stable *DSPP*-silencing in OSC2 cells suggests that DSPP may operate upstream of these protein products. Furthermore, the down-regulation of p53 indicates that DSPP may directly, or at least remotely, regulate aspects of the cell-cycle activity.

Recent reports also suggest that MMP-mediated remodeling of extracellular matrix (ECM) is, indeed, one of several initiating events allowing OSCC cells to invade the surrounding stroma [25]. It is postulated that early expression of MMP by tumor or surrounding stromal cells facilitates the remodeling of ECM, resulting in the release of growth factors to ensure a viable nidus for primary tumor growth [26], [27]. In turn, tumor growth leads to angiogenic switch during which the balance of proangiogenic factors such as VEGF overcomes the expression of angiogenic inhibitors [28]. VEGF-mediated angiogenesis is a hallmark of OSCC progression [25], [29], and MMP-2 and MMP-9 have both been implicated in the induction of the angiogenic switch in different models [26], [27]. Although, the MMP partners of DSPP and MEPE are yet to be identified, the dynamics and implications of the SIBLING-MMP interaction in biologic systems is now one of heightened research interest. For example, a recent report indicated that *in vitro* invasiveness of some cancer cell lines was enhanced via the formation of RGD-dependent complex with MMP-2 and αVβ3 integrin [30].

The resistance of head and neck squamous cell carcinomas (HNSCC), including OSCCs, to chemotherapeutic agents such as cisplatin is well documented and remains a major challenge to effective chemotherapeutic interventions in OSCCs [15], [16]. Cisplatin is effective in only about 20% of HNSCC patients. In cancers of other systems with recorded chemotherapeutic successes with cisplatin, the mechanism of action of cisplatin adduced includes the induction of apoptosis in cancer cells [15]. The resistance of OSCC to cisplatin-induced apoptosis has been attributed to the up-regulation of EGFR in HNSCCs [15]. Our present data indicate that DSPP suppression alone, while resulting in G_0_/G_1_ arrest, does not increase tumor cell death by apoptosis in OSC2 cells. Our observation that the sensitivity of OSC2 cells to cisplatin-induced apoptosis is significantly enhanced following *DSPP*-silencing, possibly through a mechanism involving the downregulation of EGFR, is therefore both significant and novel.

The *in vivo* anti-tumor effects of DSPP–silencing, while so far demonstrated in only two mice, nevertheless, suggested a trend towards reduced tumor growth. Elaborate design beyond the scope of the present study will further verify this trend in the context of other questions.

Collectively our present data showing that *DSPP*-silencing is associated with significant alteration in notable hallmarks of oral malignancy in OSC2 cells suggest that DSPP regulates multiple pathways involving the SIBLING-partnering MMPs, VEGF, and proliferation markers critical for oral cancer local progression and metastatic spread. While a detailed study of the mechanism of DSPP-oral cancer tumorigenesis was beyond the scope of our present report, our current data provides a framework for on-going study focused on deciphering DSPP mechanistic network involved in oral cancer biology. This, in turn, is with a view to identifying target points for the design of potent biomimetics for intervention in oral cancer.

## Materials and Methods

### Human Cell lines and culture conditions

The following human cell lines used in this study, SCC25 and OSC2 (OSCC), and DOK (dysplastic oral keratinocytes) have been published, and were initially obtained from American Type Culture Collection. Mammary adenocarcinoma MCF7 cell whole lysate was purchased form Santa Cruz Biotechnology (Santa Cruz, CA; Cat. #sc-2206), while human oral keratinocyte (HOK) whole lysate was purchased from ScienCell™ Research Laboratories (cat. #2616; San Diego, CA). All cell lines were routinely cultured as monolayer in DMEM/F12 medium containing 10% FBS (Invitrogen, Carlsbad, CA) supplemented with 1% Penicillin/Streptomycin and 500 ng/ml Hydrocortisone (Sigma Aldrich, St. Louis, MO) and maintained in the presence of 5% CO_2_ humidified air at 37°C.

### Antibodies

Anti-DSPP monoclonal (LF-Mb21) and polyclonal (LF-151) antibodies as well as polyclonal antibodies to MMP-2 (LF-83), MMP-3 (LF-82), and MMP-9 (LF-84), which were kind gifts from Dr. Larry W. Fisher (NIDCR, NIH Bethesda, MD) were used for immunofluorescence, western blot, and immunohistochemistry analyses. The monoclonal antibody to DSPP (LF-21) is now available from Santa Cruz Biotechnology, Inc. (Santa Cruz, CA). Polyclonal Ki-67 Mouse (Cat # sc-15402), polyclonal p53 (cat. #sc-6243), polyclonal PCNA (cat. #sc-7902), and polyclonal VEGF (cat. # 57496) were purchased from Santa Cruz Biotechnology (Santa Cruz, CA) and used for western blot analyses.

### shRNA lentiviral particle transduction

DSP-shRNA (h) lentiviral particle (cat #sc-40500-V) is a transduction-ready pool of 3 target-specific constructs encoding 19–25nt (plus hairpin) shRNAs designed to silence DSPP gene expression. copGFP Control Plasmid (cat # sc-108083) is a transfection-ready lentiviral vector plasmid that encodes copGFP fluorescent protein in mammalian cells, and used to monitor delivery of shRNA lentiviral construct into cells thereby gauging transfection efficiency. Control shRNA Plasmid-A (cat. #sc-108060) is a negative control plasmid that encodes a scrambled shRNA sequence that will not result in specific degradation of any known cellular mRNA. All three plasmid constructs, and the transfection reagent Polybrene (Cat. # sc-134220) were purchased from Santa Cruz Biotechnology, Inc (Santa Cruz, CA). The sequences of DSP-shRNA Vector Plasmid and Control shRNA Plasmid-A are as follows:

sc-40500-V (A pool of 3 different shRNA plasmids)

• Sense: GCAAGAGAAUACCCAAGAU

• Antisense: AUCUUGGGUAUUCUCUUGC

• Sense: CGAGGGUAAUACAAGUGAA

• Antisense: UUCACUUGUAUUACCCUCG

• Sense: CCAAGAUAAGGGAAUAGAA

• Antisense: UUCUAUUCCCUUAUCUUGG

sc-108060 (Plasmid Control A)

TTCTCCGAACGTGTCACGTTTCAAGAGAACGTGACACGTTCGGAGAATTTTT

Note: all sequences are provided in 5′ → 3′ orientation.

### DSP shRNA lentival mediated transduction of OSC2 cells

A day prior to transfection, 1.5×10^4^ logarithmically growing and healthy OSC2 cells were split into four equal groups each plated in 6-well plate in antibiotic-free DMEM/F12 media supplemented with 10% serum (Mediatech Inc. VA) to achieve a 70–80% confluency overnight. The groups are the “medium-only”, “Control shRNA Plasmid-A” (scrambled sequence), “copGFP Control Plasmid”, and the experimental DSP shRNA Plasmid group. Transient transfection was carried out following the manufacturer's protocol.

Prior to transfection, cells were washed with shRNA transfection medium before adding 2 ml of medium containing 5 ug/ml Polybrene (cat. # sc-134220) to each well. Thereafter 30 ul (∼30×10^4^ particles) of lentiviral particles, equivalent to multiplicity of infection factor (MOI) 1, was added drop-wise to corresponding well and incubated overnight under normal cell culture conditions. Following incubation, medium containing polybrene was removed and replaced with normal growth medium (NGM), and the cells incubated for an additional 24 hrs under normal cell culture conditions before taking post-transfection photographs of cells.

### Establishment of DSP-shRNA stably transfected OSC2 cells

Selection of OSC2 cells stably expressing DSP shRNA DNA and the Control shRNA Plasmid-A was commenced 72 h post-transfection following the manufacturer's instructions (Santa Cruz Biotechnology, Santa Cruz CA). Briefly, growth medium was aspirated from the cells and replaced with fresh selection medium containing 3 µg/mL of puromycin (cat # sc-108071; Santa Cruz Biotecnology). Puromycin-containing medium was replaced every 2–3days with freshly prepared selection medium, and selection of stable cells expressing DSP-shRNA or Control shRNA Plasmid-A was completed aproximately 4 weeks from commencement of selection. Stable cells were expanded, harvested, and prepared for western blot and quantitative real-time PCR (Q R-T PCR) analyses.

### Semiquantitative RT-PCR

Total RNA was extracted from 1/3^rd^ of the cell pellets from each group (experimental and control) using the RNEasy Plus Mini kit (Qiagen, CA) following the manufacturer's instructions. 1 µg of total RNA was used to reverse transcribe to cDNA using the High Capacity Reverse Transcription Kit (Applied BioSystems, CA) in a conventional Thermo cycler following the manufacturer's instructions. PCR reaction was performed using the Promega's GoTaq Green MasterMix reagent (cat. #M7122; Promega, Madison WI) and the DSPP primer pair from Santa Cruz Biotechnology (cat # 40500PR; Primer design amplified a 519 base amplicon from NM_014208. Exact primer sequences are proprietary and not available from company). 100 ng of cDNA was used for the 25 µl PCR reaction, and GAPDH was used as a normalizing control. PCR products were then electrophoresed on a 2% agarose gel in Tris-acetate-EDTA (TAE) buffer, stained with ethidium bromide, and photographed. Densitometric analysis of DSPP mRNA levels was performed.

### Quantitative Real-Time PCR (Q RT-PCR) analysis

From total RNA isolated from cell extracts, reverse transcription to cDNA was performed. The SybGREEN qPCR primers for DSPP (cat. #HP210697) and GAPDH (cat.#HP205798), purchased from Origene (Rockville, MD), and the SybGREEN Mastermix purchased from Bio-Rad (cat.#170-8880; Hercules, CA) were used to perform the reaction. The human Primer qSTAR qpcr primer pairs against Homo sapiens gene DSPP NM_014208 had the following sequence: Forward: CAACCATAGAGAAAGCAAACGCG

Reverse: TTTCTGTTGCCACTGCTGGGAC; while the human Primer qSTAR qpcr primer pairs against Homo sapiens gene GAPDH gene had the following sequence:

Forward: GTCTCCTCTGACTTCAACAGCG

Reverse: ACCACCCTGTTGCTGTAGCCAA

Reactions were carried out on the Real Time PCR - 7300 model instrument from Applied Biosystems following the manufacturer's protocol. A total reaction volume of 25 ul was prepared using 1 ul of 25X primer, 12.5 ul of 2X Mastermix, 100 ng cDNA and deionised water added to the desired volume. Data was analyzed using the Comparative Ct method (ΔΔCt) method for Relative Quantification (RQ). Here, ΔCt was calculated as Ct(GOI)-Ct(Control), where GOI is the Gene of Interest (DSPP) and the Control is GAPDH. ΔΔCt was calculated as ΔCt(GOI)- ΔCt(NC), where NC is the scrambled shRNA control (shC). Percent of gene remaining was calculated as 2exp-1ΔΔCt. Finally, percentage knockdown was determined from percentage of residual gene.

### Western blot-analysis

Total cell lysates prepared from 2/3^rd^ of cell pelletes from each group (experimental and controls) were resolved on SDS–PAGE gel using the Mini-Protean Tetra Cell unit at 100 V (Biorad, CA) by standard protocol. Briefly, 25 µg of protein was loaded in each lane and resolved on the appropriate percentage polyacrylamide gels before transferring to polyvinylidene (PVDF) membranes (Millipore, MA) at 100 mA constant current for 75 mins in the Biorad Transfer unit. The membrane was thereafter incubated in blocking solution consisting of 1∶1 diluted Sea Block Blocking solution (LI-COR, Biosciences, Lincoln, Nebraska) in 1x PBS, for 1 hour at RT. Membrane was then incubated in primary antibody (e.g. anti-monoclonal DSPP antibody, LF-MB21) and anti-β-actin monoclonal antibody (Sigma, St. Louis, MO) diluted in blocking buffer (1∶1000 and 1∶5000, respectively) overnight at 4°C before washing with 1x PBS-T for 5×5 mins. Thereafter, membrane was incubated with IRDye 600 goat anti-mouse secondary antibody (L-ICOR Biosciences) at 1∶5000 dilution for 1 hr at RT, washed with 1x PBS-T 4×5 mins, and with 1x PBS for 5 mins. Signal was detected using the Infrared LI-COR Imaging system (LI-COR Biosciences).

### Transwell migration and invasion assays

The motility and invasiveness of *DSPP*-silenced L2 cells and controls were evaluated using a modified Boyden Chamber assay. The Chamber consisted of a 24-well transwell chambers with upper and lower culture compartments separated by uncoated polycarbonate membranes with 8-µm sized pores (Costar 3422, Corning Inc., NY). The polycarbonate membranes remained uncoated in chambers used for the migration assay, whereas the membranes in chambers used for the invasion assays were coated with 100 µl of 100 µg/ml of Matrigel™ (Collaborative Biomedical Products, Bedford, MA) prior to the invasion assay. Prior to plating cells into the transwells, DMEM–0.1% BSA was incubated in the top chamber of each transwell at 37°C for 1 h in order to saturate non-specific binding sites. Cells were then resuspended in growth media (5×10^4^ cells/100 µl) following trypsinization before plating into the upper compartment of the migration or invasion chambers. DMEM–10% FBS was placed into the bottom compartment to act as a chemoattractant. After 24 h (for migration) and 48 h (for invasion) incubation at 37°C in 5% CO_2_ humidified air, the cells were incubated in Calcein-AM (molecular Probes) for another 1 h at 37°C before removing the remaining cells in the upper compartment surface of the membrane with a cotton swab. Cells that migrated through the pores sticking to the under surface of the membrane were fixed with 3.7% paraformaldehyde before staining with 0.2% crystal violet dye. Quantitative estimates of the number of migrated/invaded cells were carried out through colorimetric analysis with a microplate reader (absorbance at 590 nm). Assays were carried out in triplicate and results expressed as mean ± SD. Photomicrographs of representative results were taken as shown in the Results section.

### MTT assay

For MTT [3-(4,5-dimethylthiazol-2-yl)-2,5-diphenyl-tetrazolium bromide] assay methods, cells were seeded in 96-well plate (Costar, Corning Inc., NY) at a density of 2×10^3^/well. Cell growth was assessed at days 1 through 6, each day by staining with 100 µl/well of sterile MTT solution (0.5 mg/ml, Sigma Chemical Co., St Louis, MO) for 4 h at 37°C. Thereafter, cell were incubated for 5 min in 100 µl/well of Tris-Formalin solution before washing each well with 200 µl of double distilled water (ddH_2_O). 100 µl of DMSO was then added to each well, and vortexed (on lowest speed setting) for one minute. Absorbance value was measured on a microplate reader (BioRad) at 562 nm using 630 nm as reference. Assays were performed in triplicate and data are expressed as mean ± SD.

### Colony-formation assay

Cells (1000/well) were suspended in 0.35% Bactoagar and were plated onto a layer of 0.75% Bactoagar in DMEM-F12 containing 5% FBS in 6-well tissue culture plates (Corning, Corning, NY). The agar-containing cells were allowed to solidify overnight at 37°C in 5% CO_2_ humidified atmosphere. Additional DMEM-F12+5% FBS was overlaid on the agar and the cells allowed to grow undisturbed for 3weeks. Visible colonies (>50 cells) were counted with the aid of a dissecting microscope.

### Cell cycle and apoptosis analyses by flow cytometry

L2 cells and controls were harvested after tripsinization, fixed with cold 70% ethanol, and stored at 4°C pending cell cycle analyses. L2 cells were treated with 50 uM cisplatin for 24 h, washed in 1XPBS before fixing in 70% ethanol overnight. Prior to cell cycle analysis, cells were resuspended in PBS containing RNase A (20 ug/mL, Sigma) and 20 µg/ml propidium iodide (PI; Sigma), and then incubated at 37°C for 30 min before analyses for PI fluorescence intensity using the Becton Dickinson FACsort flow cytometer (Becton-Dickinson, Temse, Belgium). A total of 10,000 events were counted for each sample group. The relative proportions of cells in the G_0_/G_1_, S, and G_2_/M phases of the cell cycle were analyzed using FlowJo software (Tree Star, Ashland, OR).

For apoptosis analyses, Annexin V/PI staining of cells was carried out. Briefly, cells were washed with 1XPBS and resuspended at 2×10^6^ cell/ml in Annexin V-binding buffer before aliquoting the suspension into 100 ul/tube fractions. Thereafter, 5 ul of Annexin V FITC and 10 ul of PI buffer were added to each tube before incubating in the dark for 15 min at RT. 400 ul of 1x Annexin V-binding buffer was then added to each tube and flow cytometric analysis carried out within one hour. Controls consisted of unstained, Annexin-positive, and PI-positive cells.

### DNA fragmentation assay

L2 and controls previously treated with 50 umol/l cisplatin for 48 h were harvested and lysed in lysis buffer [0.5% SDS, 100 mmol/l EDTA, 10 mmol/l Tris-HCl (pH 8.00] before incubating overnight with proteinase K (20 mg/ml, Sigma) at 37°C and treated with RNase A (50 ug/ml, Sigma). DNA was then extracted and precipitated using the phenol/chloroform and ethanol method. Extracted DNA was then dissolved in TE buffer [10 mmol/l Tris-HCl, 1 mmol/l EDTA (pH 8.0)] and analyzed on a 1% agarose gel containing ethidium bromide (0.05 ug/ml, Sigma). DNA was visualized and photographed under UV light.

### Immunofluorescence

L2 cells and controls were grown in DMEM/Hams F-12 medium with 10% fetal calf serum, 100 IU/ml penicillin, 100 µg/ml streptomycin, and 5 µg/ml hydrocortisone before transferring to Lab-Tek 11 chamber slides (Thermo Fisher Scientific) at 80–90% confluence to grow overnight. For immunofluorescence labeling, each experimental and control group consisted of DSPP labeling following established protocol. Briefly, cell were washed one time with 1XPBS, fixed with 4% paraformaldhyde (PFA) for 10 min, washed 3X with PBS before permeabilizing with 0.5% Triton X (in 1XPBS) solution for 5 min at room temperature (RT). Cells were then incubated with DSPP primary antibody, overnight. After washing with PBS, cells were incubated with fluoresceine-conjugated secondary antibodies for 1 h. Thereafter, cells were washed and mounted with VectaShield mounting medium with 4′,6-diamidino-2-phenylindole (DAPI) (Vector Laboratories) and visualized at 100× magnification using a Nikon inverted fluorescent microscope equipped with deconvolution software (Slidebook 4.0, Intelligent Imaging, Denver, CO, USA). The number of cells expressing DSPP was evaluated visually by two observers in 10 random fields containing at least 200 cells each. Representative photographic images were taken using a laser-scanning confocal microscope as well as a light microscope.

### *In vivo* anti-tumor effects of DSPP-silencing in OSC2 cells

Homozygous Balb/c nu/nu athymic female mice (4–6 weeks of age) were purchased from the National Cancer Institute (Bethesda, MD) and maintained at 22–24°C under pathogen-limiting conditions as required. All animal manipulations were carried out in a laminar airflow biological safety cabinet. Cages, bedding, and food were autoclaved before use. Sterile water was provided ad lib. Five million exponentially growing DSPP-silenced OSC2 cells (L2) and their scarmbled sequence (shC) control counterpart were mixed in matrigel and media before injecting intraperitoneally into left and right flanks of the 2 mice, respectively. Thereafter tumor sizes were monitored and measured weekly for 6 weeks. Volume of the tumor was calculated using the formula (1/2)*A*B*B, where “A” is the length of tumor and “B” breadth of tumor. A scatter graph of tumor volume (mm3) versus weeks was generated as Supplementary Figure S1. At the end of the experimental period, animals were sacrificed, and tumors dissected for Histology.

### Immunohistochemistry

Paraffin block sections (5 µm) from L2– and shC-derived tumors were immunostained for DSPP using the *Nemesis 7200* automated system (BioCare; San Francisco, CA) supplied with Super-Picture-Perfect Broad-Spectrum HRP-Polymer and Single-Solution-AEC reagents from Zymed Lab Inc. (San Francisco, CA). Immunostaining was carried out using established and published protocol from our laboratory [9], [10]. For negative controls we substituted non-immune mouse IgG control (Zymed) in place of primary antibody (LFMb21). Photographic representative results for each case were captured using the Axioplan 2 Universal microscope equipped with an Axiovision camera and program (Carl Zeiss Gmbh, Jena, Germany).

### Statistical analysis

Data are expressed as mean ± SD. Statistical analysis was performed using SigmaStat Version 3 (Systat software, Point Richmond, CA) and SAS 9.1 (SAS Institute, Inc., Cary, NC). Due to the skewed nature of the data, the Kruskal-Wallis test and Dunn multiple comparisons were used any time more than two groups were compared. Least-squares regression through the origin was used to describe the relationship between the degree of DSSP silencing and the level of suppression of each of the three SIBLING-partnering MMPs, and to compare this relationship between pro-MMP and cleaved MMP. The criterion for significance was *P*<0.05 for all statistical tests.

## Supporting Information

Figure S1 In vivo anti-tumor effects of DSPP-silencing in OSC2 cells. (A) The anti-tumor effect of DSPP-silencing in OSC2 cells shows small tumor size (volume) for L2 derived tumor (left flank) compared with shC control derived tumors (right flank). Tumor size was measured weekly over a period of 6 weeks. (B) Histologic evaluation of hematoxylene and eosin (H&E) sections showing well differentiated and aggressive squamous cell carcinoma (arrow) associated with the shC (control) derived tumors. (C) Less-differentiated L2 derived tumors formed small shrinking islands (*) and tumor necrosis compared with shC tumors (B). (D) Immuostain for DSPP with LF-Mb21 antibody verified significant DSPP reduction in L2 derived tumors compared with high DSPP level (brown stain) in shC derived tumors (E). (F) A representative pre-immune IgG negative control. (G) Scatter plot of growth trend for L2 derived tumors (red) and the shC derived tumors (blue) showing slowed tumor development and volume for L2 tumor compared with shC tumors over a period of 6 weeks.
(2.39 MB TIF)
