# Supplementary figures and images for: Dentin Sialophosphoprotein (DSPP) Gene-Silencing Inhibits Key Tumorigenic Activities in Human Oral Cancer Cell Line, OSC2
Source: PLoS One. 2010 Nov 12;5(11):e13974. doi: 10.1371/journal.pone.0013974 (PMC2980487; doi:10.1371/journal.pone.0013974)

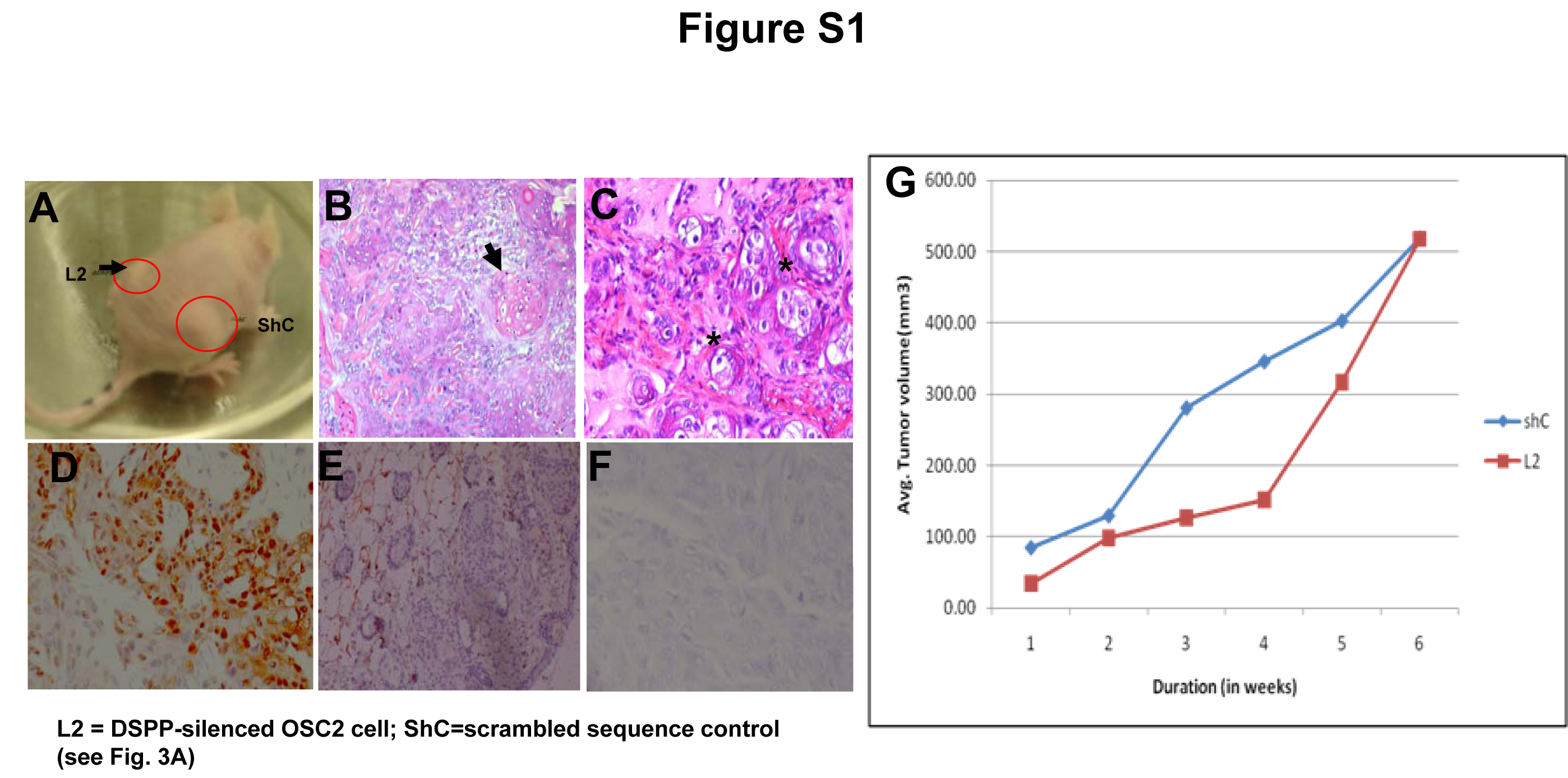

Supplement: Figure S1 — In vivo anti-tumor effects of DSPP-silencing in OSC2 cells. (A) The anti-tumor effect of DSPP-silencing in OSC2 cells shows small tumor size (volume) for L2 derived tumor (left flank) compared with shC control derived tumors (right flank). Tumor size was measured weekly over a period of 6 weeks. (B) Histologic evaluation of hematoxylene and eosin (H&E) sections showing well differentiated and aggressive squamous cell carcinoma (arrow) associated with the shC (control) derived tumors. (C) Less-differentiated L2 derived tumors formed small shrinking islands (*) and tumor necrosis compared with shC tumors (B). (D) Immuostain for DSPP with LF-Mb21 antibody verified significant DSPP reduction in L2 derived tumors compared with high DSPP level (brown stain) in shC derived tumors (E). (F) A representative pre-immune IgG negative control. (G) Scatter plot of growth trend for L2 derived tumors (red) and the shC derived tumors (blue) showing slowed tumor development and volume for L2 tumor compared with shC tumors over a period of 6 weeks. (2.39 MB TIF) [file pone.0013974.s001.tif]
